# Surgical Strategy for Isolated Caudate Lobectomy: Experience with 16 Cases

**DOI:** 10.1155/2014/983684

**Published:** 2014-07-01

**Authors:** Gendong Tian, Qiong Chen, Yuan Guo, Mujian Teng, Jie Li

**Affiliations:** ^1^Hepatobiliary and Liver Transplantation Department, The Affiliated Qianfoshan Hospital, Shandong University, No. 16766 Jingshi Road, Jinan, Shandong 250014, China; ^2^General Surgery Department, The First People's Hospital of Jinan City, No. 132 Minghu Road, Jinan, Shandong 250014, China

## Abstract

*Introduction*. Surgical resection is the most effective treatment for neoplasm in the caudate lobe. Isolated caudate lobectomy is still a challenge for hepatobiliary surgeons. No widely accepted surgical strategy for the procedure has been developed yet. *Objective*. To get a better understanding of isolated caudate lobectomy and to optimize the procedure. *Materials and Methods*. 16 cases of isolated caudate lobectomy were reviewed to summarize the surgical experience. *Results*. All the 16 cases of isolated caudate lobectomy were carried out successfully, among which left side approach was adopted in two cases (12.5%), right side approach in three cases (18.75%), and both sides approach in 11 cases (68.75%). No severe complications occurred. *Conclusion*. The majority of neoplasms confined to the caudate lobe can be resected safely by left and right side approach with proper anatomic surgical procedure, usually in the sequence of mobilization, outflow control, inflow control, and division of the hepatic parenchyma. Fully mobilizing the caudate lobe from the inferior vena cava (IVC) is of great importance. Division of the retrohepatic ligament and the venous ligament facilitated the procedure.

## 1. Introduction

The caudate lobe lies deep in the liver, between the hepatic hila and the retrohepatic inferior vena cava (IVC), and is adjacent to the major hepatic veins in its upper part. Although the caudate lobe constitutes only a small part of the whole liver, it has the same histologic structure and the same incidence of developing benign and malignant neoplasms as other hepatic segments in proportion to their volume. Percutaneous ethanol injection and radiofrequency ablation (RFA) for tumors in the caudate lobe are difficult to be carried out because of their spatial peculiarity [1]. Multiple bilateral blood supplies from hepatic artery and portal vein make transcatheter arterial chemoembolization (TACE) less effective for malignant tumors in the lobe than those in the main lobes. Surgical resection is left the only radical solution for symptomatic benign tumors and malignant tumors confined to the lobe. Isolated caudate lobectomy, a parenchyma-sparing procedure, is still a challenge for hepatobiliary surgeons, especially in cirrhotic patients. Relatively safe and reliable surgical techniques for the procedure have not been developed thoroughly. 16 cases of isolated caudate lobectomy in our departments from January 2010 to December 2013 were reviewed to optimize the operation.

According to Kumon's nomenclature [2], the caudate lobe consists of 3 portions: the Spiegel lobe (i.e., Couinaud's segment I), the paracaval portion (i.e., Couinaud's segment IX [3]), and the caudate process. The Spiegel lobe locates behind the lesser omentum, to the left of the retrohepatic IVC. The paracaval portion, which is attached to the anterior surface of the retrohepatic IVC by the retrohepatic ligament and the short hepatic veins, lies to the right of the Spiegel lobe. The upper tip of the caudate lobe extends behind the major hepatic veins. The caudate process, the smallest part of the three, is a thin tongue-like projection between the IVC and the portal vein. Kumon's definition is adopted here for better understanding. Isolated caudate lobectomy is to remove either part or total of the lobe surgically (i.e., isolated partial or complete caudate lobectomy).

Hasegawa et al. classified hepatocellular carcinomas spread from the caudate lobe into five types [4], which were frequently adopted to describe all neoplasms that originated in the caudate lobe. They were as follows:

1. type 1 lesions: lesions in the upper part of the Spiegel lobe;
2. type 2 lesions: lesions in the lower part of the Spiegel lobe;
3. type 3 lesions: lesions in the paracaval portion;
4. type 4 lesions: lesions in the caudate process;
5. type 5 lesions: lesions spread from the whole caudate lobe.

## 2. Patients and Methods

### 2.1. Patients

16 cases of isolated caudate lobectomy were performed for neoplasms confined to the caudate lobe, including seven cases of hepatocellular carcinoma (7/16, 43.75%), four cases of hepatic cavernous hemangioma (4/16, 25%), one case of hepatocellular adenoma (1/16, 6.25%), one case of inflammatory pseudotumor (1/16, 6.25%), one case of hepatic hamartoma (1/16, 6.25%), one case of mixed hepatocellular carcinoma and cholangiocellular carcinoma (1/16, 6.25%), and one case of metastatic colonic cancer (1/16, 6.25%). Hepatitis B virus surface antigen was positive in all the seven cases of hepatocellular carcinoma and in the case of mixed hepatocellular carcinoma and cholangiocellular carcinoma, respectively, and hepatitis B virus surface antibody was positive in the case of hepatocellular adenoma. The tumors were measured in the maximum diameter from 2 cm to 12 cm (4.91 cm in average). According to Hasegawa et al.'s classification [4], there were one case of type 1 lesions, three cases of type 2 lesions, three cases of type 3 lesions, six cases of type 4 lesions, and three cases of type 5 lesions (Table 1).

### 2.2. Surgical Procedure for Isolated Caudate Lobectomy

Isolated resection of the caudate lobe consisted of four major steps: mobilization of the lobe, outflow control by dividing the short hepatic veins behind the lobe, inflow control by dividing the portal triads to the lobe, and division of the hepatic parenchyma between the caudate lobe and the main liver. Left side approach, or right side approach, or both left and right sides approach were adopted in the operation. The sequence of the four steps and the surgical approach alternated according to the tumor's location, size, texture, and nature.

#### 2.2.1. Approaches to the Caudate Lobe

**Figure 1:**
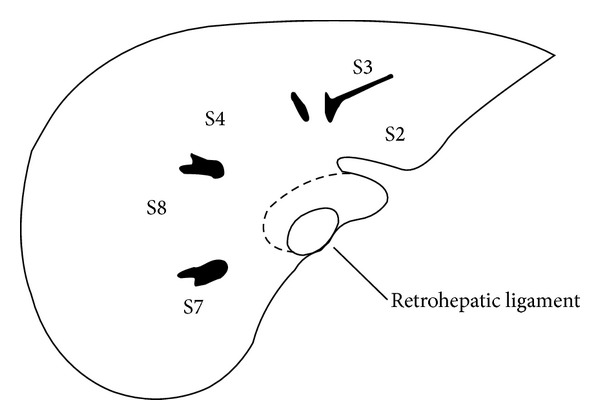
Schematic cross-sectional diagram of the caudate lobe, showing the retrohepatic ligament and the relationship between the caudate lobe and the main liver.

*Left Side Approach.* Left side approach was adopted for resection of small masses that originated in the Spiegel lobe, especially type 2 lesions. After entering the abdominal cavity through a reversed L-shaped incision on the right upper quadrant of the belly, the round, falciform, left triangular, left coronary and hepatogastric ligaments were separated. The hepatoduodenal ligament was retracted to the right to expose the Spiegel lobe. The retroperitoneum covering the left wall of the retrohepatic IVC was incised to free the left margin of the Spiegel lobe. The retrohepatic ligament was divided when necessary (Figure 1). The venous ligament was divided to free the upper tip of the caudate lobe [5]. Then, the Spiegel lobe was easily elevated from the retrohepatic IVC to expose the short hepatic veins to direct view as they were divided and ligated caudal cranially. The left portal hilum was lifted ventrally to expose the portal triads to the Spiegel lobe, which were divided and ligated subsequently. The liver parenchymal bridge was transected by CUSA.

*Right Side Approach.* Right side approach was adopted for resection of small masses that originated in the caudate process. After entering the abdominal cavity through a reversed L-shaped incision, the round, falciform, right triangular, right coronary and hepatorenal ligaments were divided in turn. The right adrenal gland was detached from the liver with care. The retroperitoneum covering the infrahepatic IVC was incised. The liver was elevated from the retrohepatic IVC and rotated to the left to show the short hepatic veins, which were divided and ligated in a cranial direction. The possible inferior right hepatic vein (IRHV), which often enters the IVC near the right adrenal gland vein, was divided also. The retrohepatic ligament was divided when necessary from the left lateral surface of the IVC through an incision on the lesser omentum. The hepatoduodenal ligament was retracted to the left to expose the caudate process. If the portal triads to the caudate process could be dissected easily, they would be divided in advance; in reverse, they were controlled during dividing the liver parenchyma by CUSA.

*Left and Right Side Approach.* Left and right side approach was adopted for resection of the majority of caudate masses. A reversed L-shaped incision on the right upper quadrant was adequate in most both sides approach cases. The transverse arm of the incision might be extended to the left subcostal region if necessary. The falciform ligament was dissected to the ventral surface of the suprahepatic IVC to show the loose space between the right hepatic vein and the confluence of the left and middle hepatic veins. The liver was fully mobilized by separation of all the peritoneal attachments. The right adrenal gland was detached from the liver. The retroperitoneum covering the infrahepatic IVC and the left wall of the retrohepatic IVC (referred to as Makuuchi's fascia in Japanese literature [6]) was incised caudal cranially to the superior recess of the lesser sac to expose the entire retrohepatic IVC. At this time, the suprahepatic and infrahepatic IVC were taped if needed. The venous ligament was divided near the root of the left hepatic vein. The confluence of the left and middle hepatic veins, which enters the anterior left lateral wall of the suprahepatic IVC, was encircled by passing a vascular tape through the latent space surrounded by the dorsal surface of the two hepatic veins, the ventral surface of the IVC, and the tip of the caudate lobe. The retrohepatic ligament was divided. The liver was lifted and all the short hepatic veins (including the IRHV) were taken down and suture-ligated. The hepatoduodenal ligament was loosened, and the duodenum and the pancreatic head were partly mobilized by Kocher maneuver for better exposure of large caudate masses. The portal triads to the caudate lobe were dissected and ligated. After the liver parenchyma between the caudate lobe and the main liver was transected, the tumors were removed en bloc.

#### 2.2.2. Dividing the Retrohepatic Ligament

The retrohepatic ligament attaches the Spiegel lobe to segment VI behind the retrohepatic IVC. Division of the ligament enabled the elevation of the caudate lobe from the caval vein. Usually the retrohepatic ligament is divided from the left side of the IVC. But when an enlarged Spiegel lobe embraced the IVC dorsally, it should be divided from the right side more conveniently [7].

#### 2.2.3. Dividing the Venous Ligament

The venous ligament (i.e., the Arantius' ligament) lies in the sulcus of the ligamentum venosum and connects the left portal vein to the root of the left hepatic vein. The venous ligament was divided near the left hepatic vein to partly release the tip of the caudate lobe and to facilitate the isolation of the confluence of the left and middle hepatic veins [5].

#### 2.2.4. Pringle Maneuver

The hepatic pedicle was encircled with a vascular tape for possible temporary inflow control of the liver.

#### 2.2.5. Controlling the IVC

After complete mobilization of the liver and incision of the retroperitoneum that covers the infrahepatic IVC and the left wall of the retrohepatic IVC, the suprahepatic and infrahepatic IVC were isolated and encircled with vascular tapes easily in case of possible temporary total hepatic blood occlusion.

#### 2.2.6. Dividing the Short Hepatic Veins, the Portal Triads to the Caudate Lobe, and the Liver Parenchyma

**Figure 2:**
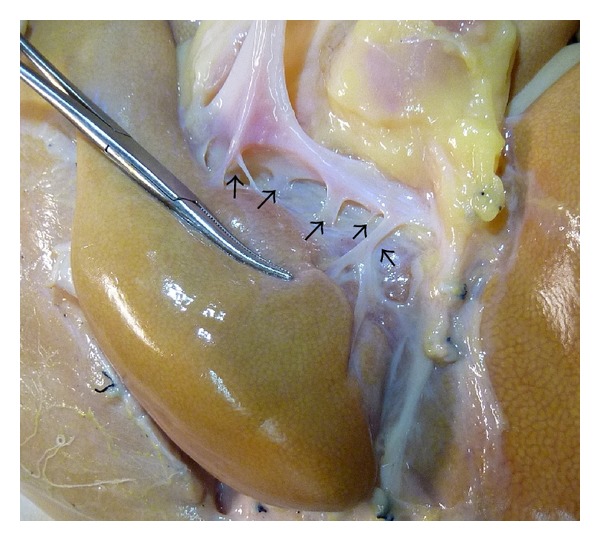
The portal branches to the caudate lobe (arrows), indicating that the portal branches to the caudate lobe originate mainly from the left hilum. Picture from unrelated specimen.

The short hepatic veins were dissected, divided, and ligated caudal cranially. These veins were best approached from the right side when there was a huge tumor [7]. Suture with transfixing stitch was obligatory for a large short hepatic vein. The whole liver was detached from the IVC besides the three main hepatic veins. The portal hila were lifted to expose the portal triads to the caudate lobe, which originate mainly from the left hilum and secondarily from the bifurcation (Figure 2) [8]. Then, the portal triads were divided and ligated serially. The parenchymal bridge between the caudate lobe and segments IV, VIII, and VII was transected by CUSA. Some small inflow vasculatures and draining vessels to the main hepatic veins, which were difficult to be dissected and ligated beforehand, were divided during transecting the liver parenchyma.

## 3. Results

All 16 cases of isolated caudate lobectomy were accomplished successfully without death and severe complications. Left side approach was adopted in two cases (2/16, 12.5%) and right side approach in three cases (3/16, 18.75%), while both sides approach in 11 cases (11/16, 68.75%). Estimated intraoperative blood loss ranged from 100 mL to 850 mL (356.25 mL in average) and transfusion varied from 0 to 800 mL (137.5 mL in average). Pringle maneuver was adopted in six cases for temporary inflow control of the liver (occlusion time ranged from 6 min to 13 min). The confluence of the left and middle hepatic veins (and the right hepatic vein in two cases) was taped in five cases for regional outflow control. The suprahepatic and infrahepatic IVC were encircled with vascular tapes in two cases, but neither needed total hepatic blood occlusion of the hepatic hila and the IVC at the same time. Small leakage in the retrohepatic IVC or the major hepatic veins was encountered in five cases, which was repaired with Prolene suture. Total operative time ranged from 150 min to 270 min (211.25 min in average). Ascites and/or right pleural effusion developed after operation in four cases. No bile leakage was encountered, which was reported as the major complication after isolated caudate lobectomy [6]. The seven cases of hepatocellular carcinoma and the case of mixed hepatocellular carcinoma and cholangiocellular carcinoma were followed up from 6 to 28 months, with one death case due to liver failure and upper gastrointestinal hemorrhage in the 26th month after operation and two cases of recurred hepatocellular carcinoma in the main liver, who thereafter received TACE, percutaneous RFA, and systemic chemotherapy. Patients with benign caudate diseases all survived in good condition.

## 4. Discussion

Isolated caudate lobectomy demands elaborate anatomy of the liver and exquisite skill of operation. The regular hepatectomy techniques are seldom employed in the operation [8]. The surgical strategy chosen for each patient must be based on the patient's idiographic situation. In the 16 cases of isolated caudate lobectomy, left side approach was adopted in two cases, right side approach in three cases, and combined left and right sides approach in 11 cases. Left side approach is adopted for small lesions in the lower part of the Spiegel lobe and right side approach for small lesions in the caudate process. Combined left and right sides approach is recommended for the majority of neoplasms in the caudate lobe [5, 9], especially for those that are bigger than 4 cm in diameter, those originating in the paracaval portion or in the whole caudate lobe, or those that are thought to be malignant tumors, which require total caudate lobectomy for clearance of the tumors. Symptomatic and continuously enlarging hepatic cavernous hemangioma also requires total caudate lobectomy before it reaches a nonresectable size. In fact, complete caudate lobectomy is technically easier and controllable than partial resection of the lobe [8]. Loosening the hepatoduodenal ligament and performing the Kocher maneuver help to expose the caudate lobe better and provide more space for the operation. When a bulky caudate tumor protrudes into the space between the right and middle hepatic veins or compresses the major hepatic veins severely, the anterior transhepatic approach should be employed in addition [10, 11] or combined lobectomy should be adopted.

Thorough medical imaging study of CT or/and MRI scan and type-B ultrasonography before operation is mandatory to illuminate the anatomic relationship between the masses and the hepatic hila, the major hepatic veins, and the retrohepatic IVC and to rule out metastases of malignant tumors. MRI scan provides more helpful information concerning the main vessels and the bile ducts. Although thrombosis in the portal vein is not thought to be a contraindication for isolated caudate lobectomy [12], ruling out thrombus in the portal vein and the IVC is important for the choice of strategy of treatment. Usually it is easy to dissect and divide the short hepatic veins along a tumor-free plane on the ventral surface of the retrohepatic IVC unless the tumor has involved the caval wall substantially [8]. Unlike the high incidence of thrombosis in the portal vein or the retrohepatic IVC in advanced hepatocellular carcinoma in the main liver or hilar cholangiocarcinoma which have involved the caudate lobe, the incidence of thrombosis is much lower than that expected for caudate tumors.

Dissection and division of the short hepatic veins are the major difficulty in isolated caudate lobectomy [9]. Complete mobilization of the liver is essential. Exposure of the entire course of the retrohepatic IVC by incising the covered retroperitoneum is of utmost importance. The superior recess of the lesser sac extends rightwards behind the Spiegel lobe and the retrohepatic IVC. The suprahepatic IVC is readily taped after the incision of this part of retroperitoneum. The short hepatic veins are first divided before the portal triads to separate the caudate lobe from the IVC, in case of removing the lobe quickly after temporary hemostasis of a possible huge bleeding from a parenchymal laceration or a tear in the major hepatic veins by Pringle maneuver. The length of the short hepatic veins is in proportion to their diameter reversely. Small short hepatic veins are easily isolated and divided. Large short hepatic veins should be tied and sutured on the hepatic side. On the caval side, the large dissected end should be repaired with Prolene suture after applying a side-wall vascular clamp. The largest short hepatic vein drains the bulkiest part of the caudate lobe, which locates predominantly in the middle of the Spiegel lobe and secondarily around the central part of the paracaval portion. The IRHV usually has a relatively large diameter also. A medium-sized short hepatic vein that drains the tip of the caudate lobe to the left hepatic vein or the nearby IVC is sometimes found, which should be ligated with care so as not to be torn. Small crevasse in the IVC is repaired with ease after hemostasis by finger press. A broken hepatic vein is repaired more conveniently after the caudate lobe is moved out as it is facilitated by Pringle maneuver. If combined with occlusion of the confluence of the left and middle hepatic veins (and sometimes the right hepatic vein), a more clear operative field is presented with less possibility of air embolism. In contrast, inflow control by dividing the portal triads to the caudate lobe is somewhat easy.

The caudate lobe attaches to the IVC circumferently by retrohepatic ligament and adheres to the caudal side of left liver by the venous ligament. When the caudate lobe is small or when the tumor locates mainly in the caudate process, the retrohepatic ligament is divided from the left side easily. But when the caudate lobe is enlarged, especially when it embraces the retrohepatic IVC dorsally with a bulky Spiegel lobe, the retrohepatic ligament should be divided from the right side [7]. Division of the retrohepatic ligament makes it much easier to elevate the caudate lobe from the ventral surface of the retrohepatic IVC, facilitates the exposure and division of the short hepatic veins, and contributes to the isolation of the main hepatic veins likewise [13]. The IRHV should be divided beforehand, which will facilitate the management of the other short hepatic veins from the right side. The division of the venous ligament loosens the cranial polar of the caudate lobe, facilitates mobilizing the caudate lobe from the left side, provides more space for encircling the left and middle hepatic veins, and minimizes the possibility of injuring the veins [5].

Hepatocellular carcinoma usually occurs after hepatitis B in China, no matter where the first tumor appears. After the original hepatic cancer in the caudate lobe is resected, the tumor may easily recur in the main liver lobes. Close follow-up of these patients at an interval of one to two months is necessary. Chemotherapy, transarterial embolization or chemoembolization, percutaneous RFA, and alcohol injection are adopted to reduce the possibility of recurrence or to treat a recurred tumor. Many authors have reported comparable (or even better) survival rate after isolated resection of caudate hepatocellular carcinoma with those in the main liver [1, 14, 15], which suggests that isolated caudate lobectomy for hepatocellular carcinoma in the caudate lobe is practical.

In summary, left and right side approach is adopted for isolated resection of most neoplasms in the caudate lobe. Mobilizing the whole caudate lobe is more important than enucleating the tumor itself in the operation. Considering the patients' safety as well as eradication of the diseases, the best surgical strategy is the one that is uncomplicated and easy to be mastered by most hepatobiliary surgeons, with fewer traumas and complications. And under the principle of mobilizing the caudate lobe firstly, controlling the outflow and then inflow vessels secondly, and dividing the liver parenchyma thirdly, it is advised that the relatively easier and safer step should be carried out first. Likewise, two steps can be carried out alternately. For example, some short hepatic veins can be divided while dividing the retrohepatic ligament; and some portal triads to the caudate lobe can be divided while dividing the liver parenchyma. Patients with malignant tumors should be followed up regularly after operation for recurrence and for adjuvant therapy.

## Figures and Tables

**Table 1:** Diagnosis, size, location, and surgical approach of 16 cases of isolated caudate lobectomy.

Case number	Diagnosis	Size (cm)^#^	Location∗	Approach
1	Hepatocellular carcinoma	3	Type 4	R
2	Hepatocellular carcinoma	3.5	Type 3	L and R
3	Hepatocellular carcinoma	6	Type 2	L and R
4	Hepatocellular carcinoma	4	Type 2	L and R
5	Hepatocellular carcinoma	2	Type 1	L
6	Hepatocellular carcinoma	5	Type 5	L and R
7	Hepatocellular carcinoma	4.5	Type 4	L and R
8	Hepatic cavernous hemangioma	5	Type 3	L and R
9	Hepatic cavernous hemangioma	8.5	Type 5	L and R
10	Hepatic cavernous hemangioma	7	Type 5	L and R
11	Hepatic cavernous hemangioma	5.5	Type 4	L and R
12	Hepatocellular adenoma	3	Type 2	L
13	Inflammatory pseudotumor	2	Type 4	R
14	Hepatic hamartoma	12	Type 4	L and R
15	Mixed hepatocellular carcinoma and cholangiocellular carcinoma	4.5	Type 3	L and R
16	Metastatic colonic cancer	3	Type 4	R

L: left side approach; R: right side approach; L and R: left and right side approach.

^#^Indicated in the maximum diameter.

*According to Hasegawa et al.'s classification [4].
